# Transduction of Single Nanomechanical Pillar Resonators by Scattering of Surface Acoustic Waves

**DOI:** 10.1021/acs.nanolett.3c00605

**Published:** 2023-05-11

**Authors:** Hendrik Kähler, Holger Arthaber, Robert Winkler, Robert G. West, Ioan Ignat, Harald Plank, Silvan Schmid

**Affiliations:** † Institute of Sensor and Actuator Systems, TU Wien, Gusshausstrasse 27−29, 1040 Vienna, Austria; ‡ Institute of Electrodynamics, Microwave and Circuit Engineering, TU Wien, Gusshausstrasse 25, 1040 Vienna, Austria; § Christian Doppler Laboratory for Direct-Write Fabrication of 3D Nanoprobes (DEFINE), Institute of Electron Microscopy and Nanoanalysis, Graz University of Technology, Steyrergasse 17, 8010 Graz, Austria; ∥ Institute of Electron Microscopy and Nanoanalysis, Graz University of Technology, Steyrergasse 17, 8010 Graz, Austria; ⊥ Graz Centre for Electron Microscopy, Steyrergasse 17, 8010 Graz, Austria

**Keywords:** Nanomechanical Pillar Resonators, Surface Acoustic Waves (SAWs), Resonant Scattering, Nanomechanical Resonators, Nanoelectromechanical Systems (NEMS), Nanomechanical Sensing

## Abstract

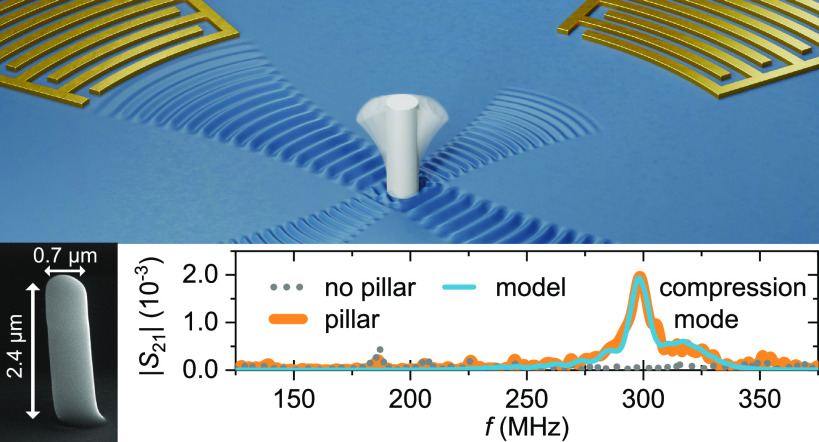

One of the challenges of nanoelectromechanical systems (NEMS) is the effective transduction of the tiny resonators. Vertical structures, such as nanomechanical pillar resonators, which are exploited in optomechanics, acoustic metamaterials, and nanomechanical sensing, are particularly challenging to transduce. Existing electromechanical transduction methods are ill-suited as they put constraints on the pillars’ material and do not enable a transduction of freestanding pillars. Here, we present an electromechanical transduction method for single nanomechanical pillar resonators based on surface acoustic waves (SAWs). We demonstrate the transduction of freestanding nanomechanical platinum–carbon pillars in the first-order bending and compression mode. Since the principle of the transduction method is based on resonant scattering of a SAW by a nanomechanical resonator, our transduction method is independent of the pillar’s material and not limited to pillar-shaped geometries. It represents a general method to transduce vertical mechanical resonators with nanoscale lateral dimensions.

Micro- and nanomechanical pillar resonators are extremely versatile due to their vertical structure and capability to be arranged in dense arrays. Pillar resonators allow for the mass detection of nanoparticles,^1,2^ the sensing of forces,^3−5^ the strong confinement of photons and phonons,^6,7^ and the manipulation of quantum dots^8−10^ and surface acoustic waves (SAWs),^11−16^ which are both exploited for quantum information processing.^17,18^ However, many of the common electrical transduction methods used for horizontally oriented nanoelectromechanical systems (NEMS) are not convenient for vertical pillar resonators, such as piezoresisitive,^19,20^ piezoelectric,^21,22^ electrothermal,^23^ and magnetomotive transduction.^24,25^ These methods rely on electrodes directly placed on top of the mechanical resonator, which cannot be done for pillars with standard lithographic fabrication techniques. That limits the feasible electrical transduction methods to capacitive transduction^26−29^ and transduction by dielectric forces.^30,31^ Both were successfully used for pillar resonators,^32,33^ but electrodes have to be placed close to the mechanical resonator for both transduction methods. In cases where such electrodes are deposited on the resonator’s substrate, the distance between the electrodes and the mechanical resonator is typically on the order of the smallest lateral dimension of the resonator.^29,30,33^ This comes with two disadvantages for pillar resonators. First, the electrodes have approximately the same height as the pillars,^32,33^ which considerably complicates the fabrication process. Second, the pillars are not freestanding, which is unfavorable for sensing applications, such as force sensing and particle mass detection. Apart from pure electrical transduction methods, optical methods^3,12,34^ and scanning electron microscopy (SEM)^1,35^ have been used to detect the motion of single pillars. These approaches have the advantage that the pillars are freestanding, but they are difficult to integrate.

Here, we demonstrate a transduction method for single pillar resonators, which combines the advantages of electrical and optical transduction methods by using SAWs. The SAWs are launched and detected by interdigital transducers distanced hundreds of micrometers away from the pillar resonator. This enables a transduction of freestanding pillars and reduces constraints on the pillars’ fabrication process.

A schematic and a SEM image of a device used in this study are shown in Figure 1a,b. The device consists of two perpendicularly oriented interdigital transducers (IDTs) and a single pillar resonator. As a substrate, we used piezoelectric lithium niobate (LiNbO_3_) with a 128° Y-cut orientation. The pillars were deposited by focused electron beam induced deposition (FEBID),^36^ where gaseous precursor molecules are locally dissociated for deposition on the substrate surface by a focused electron beam. We used platinum metal–organic precursor molecules to grow the pillars. The IDTs, fabricated by photolithography, convert an electrical input signal to a SAW and vice versa. One of the IDTs launches a SAW to drive the pillar resonator, and the other IDT measures the SAW created by the pillar’s motion, as illustrated in Figure 1a. The IDTs are optimized for the generation and detection of Rayleigh-type SAWs. To maximize the signal strength, the electrodes of the IDTs are designed to follow the shape of the wave surface of the SAW^37−39^ that is emitted by the pillar. The distance between the electrodes of the IDTs increases with increasing distance from the focal point.^40^ This is called chirping and improves the IDTs’ bandwidth. The two IDTs of a device are designed to be equivalent, taking into account the anisotropy of the LiNbO_3_ substrate. As a consequence, the two IDTs differ in the shape of their electrodes and the distances between them. Further details to the design of the IDTs and the fabrication of the devices are given in section S3 of the Supporting Information.

**Figure 1:**
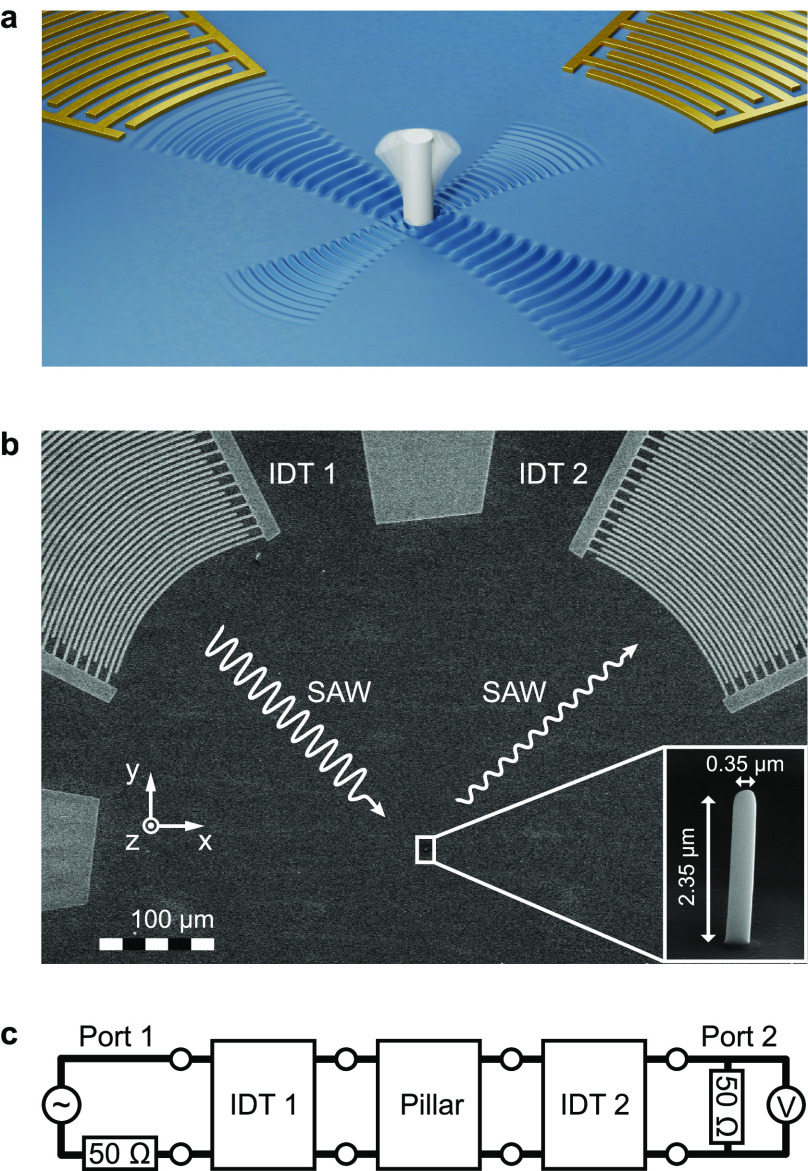
Surface acoustic wave (SAW) transduction scheme. (a) Illustration of the SAW transduction. The key components of the device are a piezoelectric substrate, two interdigital transducers (IDTs), and a pillar resonator, which are colored in blue, gold, and gray, respectively. One IDT emits a SAW to drive the pillar resonator in the center. The other IDT detects the motion of the pillar by measuring the SAW scattered by the pillar resonator at resonance. The pillar vibrates in its first bending mode. (b) Scanning electron microscope image of a device used in this study. The white, wavy lines represent the SAWs, which are launched and detected by the interdigital transducers (IDTs). (c) Equivalent circuit model. The device is represented by a cascade of two-port networks.

In the following, we discuss the results of three devices with pillars of different dimensions. We refer to the them as the thin, the midsize, and the wide pillar. The dimensions of all pillars are given in Table 1 as well as the central frequency and bandwidth of the corresponding IDTs. Both IDTs of a device are designed to have the same central frequency and bandwidth. The bandwidth of the IDTs is defined by a reduction in output power by −3 dB. Table 1 also shows the wavelengths of the SAWs emitted and detected by the IDTs at *f*_c_.

**Table 1:** Key Parameters of the Devices Used in This Study: Pillars’ Diameter *d* and Height *h*, the IDTs’ Central Frequency *f*_c_ and Bandwidth BW, and the Wavelengths λ_SAW_ of the SAWs Emitted and Detected by the IDTs at *f*_c_^a^

pillar			IDTs		
name	*d* (μm)	*h* (μm)	*f*_c_ (MHz)	BW (MHz)	λ_SAW_ (μm)
thin	0.35	2.35 ± 0.05	280	110	13.6 ± 0.7
midsize	0.70	2.40 ± 0.05	280	110	13.6 ± 0.7
wide	2.20	1.7 ± 0.1	178	70	21.3 ± 1.0

a It is not a single wavelength due to the anisotropy of the LiNbO_3_ substrate.

In the field of microwave engineering, complex circuits are often modeled by two-port networks and described by so-called scattering parameters, such as SAW devices.^40^ Scattering parameters represent ratios of outgoing to incoming normalized power waves and hence are well-suited to describe the scattering of a SAW by a pillar in an equivalent circuit model. Such a model of the SAW transduction scheme is shown in Figure 1c. The two IDTs and the single pillar are each modeled by a two-port network defined by scattering parameters. The traveling of the incoming and scattered SAWs is included in the respective IDT networks. In a cascade of two-port networks, power waves can travel back and forward between the single networks, which complicates the calculation of the overall transmission scattering parameter *S*_21_. However, it can be assumed that backscattering between the networks is minimal due to low reflection of SAWs at the IDTs and the pillar. The IDTs are chirped, and the diameters of the pillars are around a tenth of the SAWs’ wavelength or smaller at the pillars’ resonances. In this case, the overall transmission scattering parameter *S*_21_ is given by
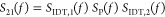
1where *f* is the frequency of the applied input signal, and *S*_IDT,i_ and *S*_P_ are the transmission scattering parameters of the IDTs and the pillar, respectively. We make a distinction between the two IDTs, since they are placed along different crystalline axes of the LiNbO_3_ substrate which results in a different electromechanical coupling of the IDTs to the substrate.^41^ However, the IDTs are designed to be equivalent, taking into account the anisotropy of the LiNbO_3_ substrate. If we assume that the electromechanical coupling of an IDT to the substrate only determines the amplitude of the transmission of an IDT, since the frequency characteristic of an IDT is mainly given by the distances of its electrodes,^40^ the transmission scattering parameters of the two IDTs are proportional to each other and *S*_21_ simplifies to
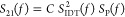
2where *C* is a constant.

The scattering of the SAW by the pillar resembles the scattering of light by resonating objects with dimensions much smaller than the optical wavelength, such as molecules or metallic nanoparticles. The scattering cross section σ_scat_ of such scattering processes is given by^42,43^
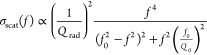
3where *f* is the frequency of the light, and *f*_0_, *Q*_0_, and *Q*_rad_ are the eigenfrequency and the total and radiation quality factor of the resonating object, respectively. Scattering cross sections describe the ratio of the scattered power to the intensity of the incident wave. In contrast, scattering parameters are defined by the square root of the incoming to outgoing power. Having this in mind, *S*_P_ is given from eq 3 as follows,
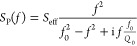
4where *S*_eff_ is an effective scattering parameter. *S*_eff_ is proportional to 1/*Q*_rad_ and is quite likely also a function of frequency. The SAW scattered by the pillar is focused by the IDT and it can be expected that the beam width of the SAW changes with frequency. Additionally, the penetration depth of the SAW into the substrate is a function of frequency.^40,44^ However, we assume *S*_eff_ to be constant in the following, since we are only interested in the narrow frequency regions around the resonances of the pillars.

It can be seen from eq 4 that the transmission scattering parameter of the pillar *S*_P_ includes the normalized frequency response of a single, weakly damped resonator. The maximum absolute value of |*S*_P_| is at the pillar’s resonance frequency *f*_res_ and consequently proportional to^45^
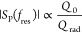
5It becomes clear from eq 5 that the pillar scatters the most when its damping is dominated by radiation losses, as expected. If this is already the case, |*S*_P_| and the overall signal strength cannot be further maximized by improving *Q*_rad_.

In addition to the transduction only by SAWs, we investigated optically the motion of the wide pillar induced by SAWs. A schematic of the optical detection setup is shown in Figure 2a. The optical signal is generated by scattering of the incident and reflected light by the lateral motion of the pillar, as demonstrated by Molina et al.^34^ The frequency responses of the wide pillar for two different laser positions on the edge of the pillar are shown in Figure 2b,c. It can be seen that the incident SAW excites two eigenmodes of the pillar: one at 160 MHz with a quality factor of *Q* = 32 and the other at 167 MHz with a quality factor of *Q* = 41.

**Figure 2:**
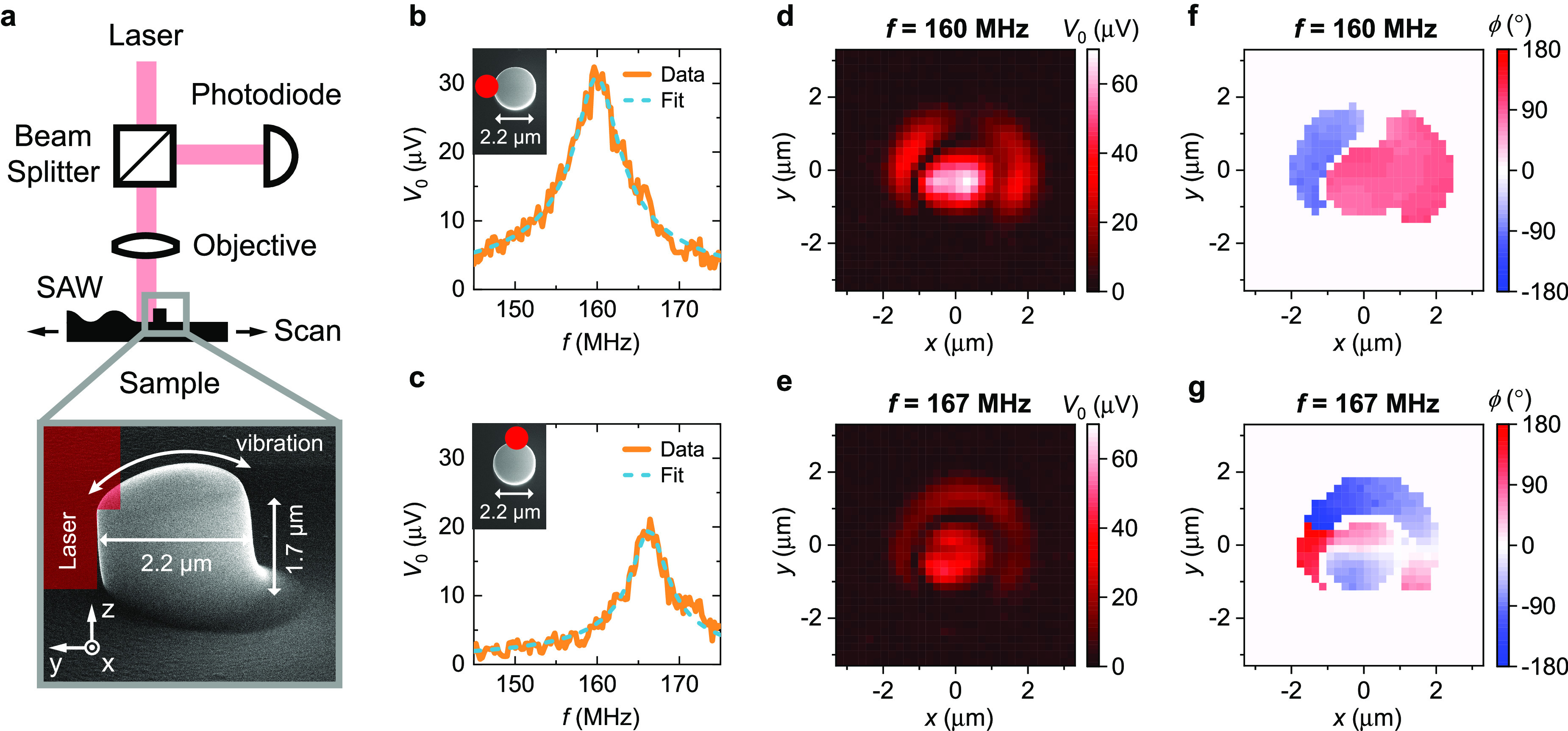
Optical detection of the motion of a pillar resonator. (a) Schematic of the optical setup and a scanning electron microscope image of the investigated pillar. The given height of the pillar is its average height. The pillar was driven by a surface acoustic wave (SAW). (b, c) Frequency response of the pillar for two different laser positions. We measured the amplitude of the photodiode’s output signal *V*_0_ at the applied SAW’s frequency. The insets show the pillar in top view. We fitted the frequency response of a driven, weakly damped harmonic oscillator to the data. (d, e) Amplitude and (f, g) phase of the optical signal for fixed frequencies as a function of the laser position with a resolution of 200 nm. For clarity, we only show the phase of the optical signal at the laser positions, where the optical signal is above the noise level. The pillar is located around the center of the maps.

To determine the type of these two modes, we measured the amplitude and phase of the optical signal for the two frequencies as a function of the laser position. The results are given in Figure 2d–g and correspond with the results of Molina et al.^34^ (see Supporting Information section S1). Two orthogonal bending modes are clearly visible: one mode vibrating along the *x*-direction and the other along the *y*-direction. Both modes show a phase difference of 180° between the opposite sides of the pillar, which is typical for bending modes. The relatively large frequency difference between the two orthogonal bending modes originates from the geometrical asymmetry of the pillar, as can be seen in Figure 2a. The pillar shows a ramp on one side of its base. The ramp is a result of a drift of the electron beam at the start of the pillar’s writing process due to charging effects and causes a reduction of the signal amplitude in negative *y*-direction, as can be seen in Figure 2e.

We compared the optical results to finite element method (FEM) simulations by simulating the eigenmodes of the wide pillar. The material properties of the pillar were based on previous studies.^38,46,47^ We set the Young’s modulus, mass density, and Poisson’s ratio to *E* = (25 ± 15) GPa, ρ = 4000 kg/m^3^, and ν = 0.38, respectively. The relatively large range of the Young’s modulus includes the possibility that the pillar experienced e-beam curing during its fabrication process.^48^ E-beam curing describes the chemical modification of FEBID-fabricated, platinum–carbon pillars that are exposed to high doses of electrons, resulting in a significant increase of the Young’s modulus.^46^ Based on the study of Arnold et al.,^46^ we expect an e-beam curing of our pillars for two reasons. First, we used larger e-beam currents of 91 pA for fabrication of the pillars compared to Arnold et al. Second, our pillars are relatively wide in comparison to the electron interaction volume^49^ so that electrons scattered horizontally in the pillar contribute to curing as well.

Apart from the pillar, we modeled the substrate as a half-sphere and defined the outer part as perfectly matched layer to mimick an infinitely large substrate. The substrate material was 128° Y-cut LiNbO_3_. We exploited the symmetry of the lithium niobate crystal^50^ and reduced the simulated domain to half of the considered domain, as shown in Figure 3a. Figure 3b,c illustrates the shape of the first-order bending and compression mode of the simulated pillar. Both bending and compression modes are actuated by a Rayleigh-type SAW due to its longitudinal and transverse motions.^38,51^ In our simulations, we focused on the pillar’s bending modes because of the optical measurements. The FEM simulations gave an eigenfrequency of (148 ± 54) MHz for the first-order bending modes and (381 ± 139) MHz for the second-order bending modes. The specified ranges indicate the minimal and maximal eigenfrequency to be expected based on the uncertainties in the Young’s modulus and the pillar’s height. The two measured bending modes of the pillar were around 160 and 167 MHz, which correspond to a Young’s modulus of the pillar of around *E* = (29 ± 5) GPa. The comparison between the simulated and measured eigenfrequencies suggests that we detected the first-order bending modes of the pillar.

**Figure 3:**
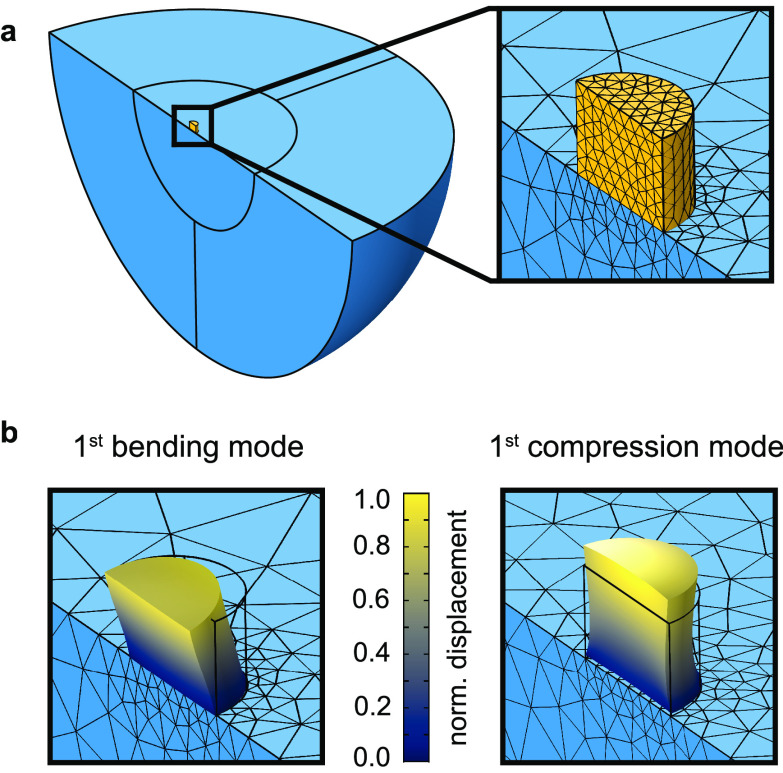
Finite element method (FEM) simulations of a single pillar resonator. (a) Geometry and mesh of the FEM simulations. The platinum–carbon pillar is colored in yellow and the lithium niobate substrate in blue. We reduced the simulated domain to half of the considered domain by exploiting symmetries. (b) Illustration of the shape of the first-order bending and compression mode of the pillar.

We also performed FEM simulations of the thin and the midsize pillar and searched for eigenmodes of both pillars with eigenfrequencies inside the frequency range of the IDTs from around 225 to 335 MHz. We set the Young’s modulus of the pillars to *E* = 29 ± 5 GPa, as determined above. We found two eigenmodes for each pillar in the frequency range of the IDTs. The thin pillar vibrates at *f*_0_ = (283 ± 30) MHz in the first-order compression mode and at *f*_0_ = (351 ± 43) MHz in the third-order bending mode. The midsize pillar vibrates around the same frequency *f*_0_ = (275 ± 28) MHz as the thin pillar in the first-order compression mode and around *f*_0_ = (219 ± 25) MHz in the second-order bending mode.

In the following, we discuss the frequency responses of the thin, the midsize, and the wide pillar measured by the SAW transduction scheme. We start with the measurements of the wide pillar. In Figure 4a two frequency responses are displayed: a measurement of the device with the wide pillar and a measurement of an identical device without any pillar. Only the device with the pillar shows a peak well above the noise level, which confirms that we measured an eigenmode of the pillar. We analyzed the frequency response of the device by fitting |*S*_21_|, as defined by eq 2, to the data. We described the scattering parameter of the pillars *S*_P_ as given by eq 4 and determined *S*_IDT_^2^ by a measurement of a device with two focused IDTs facing each other (see Supporting Information section S2). The IDTs were designed to be equivalent to the orthogonally arranged IDTs used for the pillar measurements, taking into account the anisotropy of the LiNbO_3_ substrate. We decided to fit |*S*_21_| instead of |*S*_21_|/*S*_IDT_^2^, since the normalization results in a drastically increase of the noise outside the IDTs’ frequency range. The result of the fitting is shown in Figure 4a and is in excellent agreement with the measured data. The model gives an eigenfrequency of *f*_0_ = 169 MHz and a quality factor of *Q* = 39. The comparison to the optical measurements discussed above shows that we measured the first-order bending mode of the wide pillar along the *y*-direction. However, we were not able to detect the bending mode of the pillar along the *x*-direction. A swap of emitter and receiver IDT gives the same result due to the reciprocity of the device.^40^ Hence, the bending mode in *x*-direction does not emit a significant intensity in the direction of the detection IDT and is only weakly actuated by the same.

**Figure 4:**
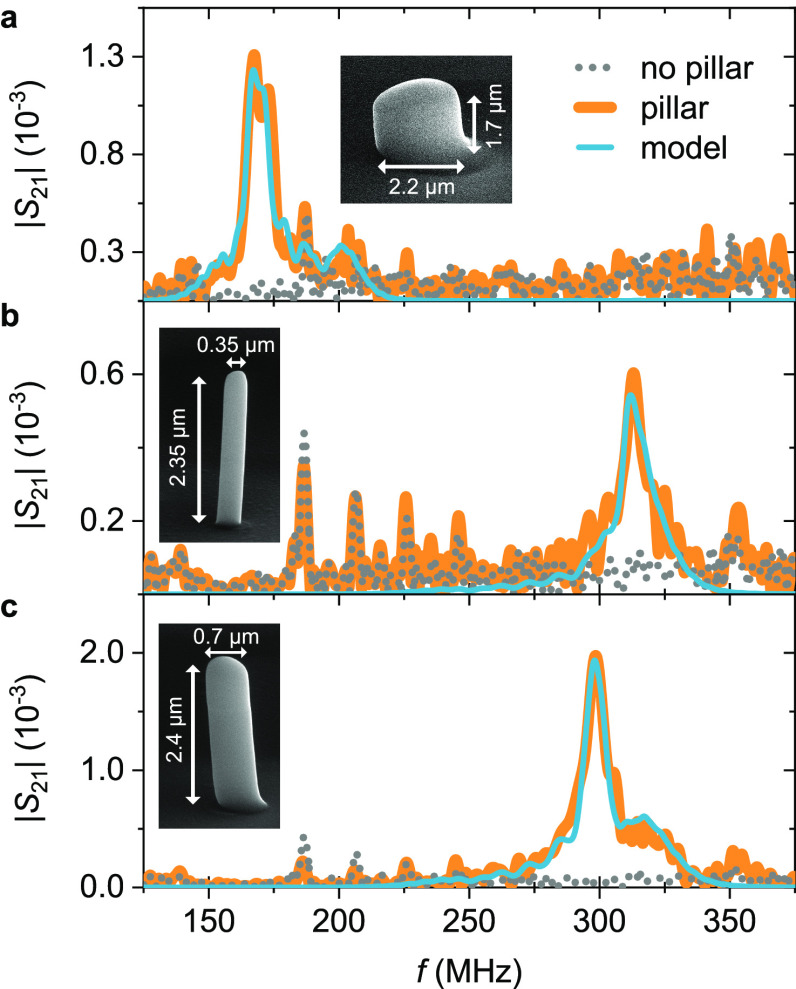
Frequency response of pillar resonators transduced by surface acoustic waves (SAW). The pillars scatter an incident SAW toward an interdigital transducer. Around the resonance of the pillars, the scattering of the incident SAW is stronger which results in an increased scattering parameter *S*_21_. Scanning electron microscope images show the geometry of the measured pillars. The widest pillar, shown in (a), vibrates in the first-order bending mode and the thinner pillars, shown in (b) and (c), in the first-order compression mode. We fitted eq 2 to the data with *S*_P_ given by eq 4.

In Figure 4b,c, the frequency responses of the thinner pillars are given in comparison to measurements of identical devices without a pillar. The results clearly show that we measured eigenmodes of the pillars. We fitted eq 2 to the data as described above. The model agrees well with the measurements and gives for the thin and midsized pillar an eigenfrequency of around *f*_0_ = 311 MHz and *f*_0_ = 298 MHz, with quality factors of *Q* = 46 and *Q* = 43, respectively. Both pillars vibrate around the same eigenfrequency despite their difference in diameter by a factor of 2. This suggests that we measured the compression mode of both pillars, since the eigenfrequency of compression modes is mainly a function of the height of a pillar and not its diameter.^45^ These results are in agreement with the FEM simulations discussed above.

The frequency responses of the devices of the thinner pillars show additional peaks to the pillars’ resonance peak, especially between 175 and 250 MHz, as can be clearly seen in Figure 4b. These peaks do not represent eigenmodes of the pillars, since they are also present in the frequency response of the device without a pillar. The peaks are also probably not electrical resonances, since we removed any electrical crosstalk from the output signal, as discussed in section S4 in the Supporting Information. We assume that these peaks originate in acoustic waves, which directly propagate from one IDT to the other by either SAWs of non Rayleigh-type or bulk acoustic waves (BAW) reflected at the bottom of the substrate.

In conclusion, we demonstrated an electromechanical transduction method for nanomechanical pillar resonators. The technique is reminiscent to darkfield microscopy but probing with SAWs. We showed that the SAW transduction method is able to actuate and detect the motion of pillars with significantly different aspect ratios. One of the pillars vibrates in its first-order bending mode, and the other two vibrate in their first-order compression mode. Our results illustrate the versatility of the SAW transduction, which originates from its working principle. The SAW transduction is based on resonant scattering of a SAW by a mechanical resonator. As a result, the SAW is not limited by the conductivity of the resonator’s material or to a specific resonator geometry. This enables adjustment of the resonator for different sensing purposes. A limit of the SAW transduction is the requirement for a piezoelectric material beneath the IDTs for SAW generation and detection, which can put constraints on the fabrication process. For the propagation of Rayleigh waves no piezoelectricity is required.

We operated at a maximum frequency of around 300 MHz. However, the SAW transduction scheme is scalable. Commercial SAW devices are usually operated at frequencies of several 10 MHz to several GHz. For example, an increase of the SAW’s frequency to 2 GHz would enable the measurement of pillar resonators with diameters and heights of around 50 and 360 nm, respectively, and a total mass of just a few femtograms, exemplifying the potential of the SAW transduction as a means to access mechanical resonators on the nanoscale. We expect that the SAW transduction method presented here enables specific applications of nanomechanical pillar resonators, e.g., for mass spectrometry or high-speed atomic force microscopy (HS-AFM). Two remaining issues of NEMS-based mass spectrometry are low mass sensitivity and throughput. A solution can be to arrange tiny NEMS resonators in 2D arrays.^52,53^ The presented SAW technique could enable the transduction of dense arrays of nanomechanical pillar resonators, since it does not rely on electrical lines close to the resonators like other transduction schemes. Additionally, with a mass of just a few femtograms, the pillars would provide a significantly improved mass responsivity compared to state-of-the-art NEMS resonators used for 2D arrays, which have individual masses in the picogram regime.^53^ State-of-the-art HS-AFM operates at frequencies of a few MHz and is limited, among other things, by the resonance frequency of the cantilever that supports the scanning tip.^54^ In comparison, SAW transduction allows the transduction of tip-like pillars in the hundreds of MHz, which corresponds to lower response times by 2 orders of magnitude. Moreover, we think that the SAW transduction scheme can facilitate developments and research in other fields that use SAWs or pillar resonators, such as quantum information technology and acoustic metamaterials.^11,18^

## Supporting Information Available

Theory of the optical detection method, measurements of the transmission scattering parameter of the IDTs |*S*_IDT_^2^|, and additional details of the device fabrication, FEM simulations, and the electrical and optical measurements (PDF)
