# Supplementary material for: Transduction of Single Nanomechanical Pillar Resonators by Scattering of Surface Acoustic Waves
Source: Nano Lett. 2023 May 11;23(10):4344–50. doi: 10.1021/acs.nanolett.3c00605 (PMC10214454; doi:10.1021/acs.nanolett.3c00605)
Supplement: Supplementary file 1 — nl3c00605_si_001.pdf [file nl3c00605_si_001.pdf]

Supporting Information for: Transduction of single nanomechanical pillar resonators by scattering of surface acoustic waves

Hendrik Kähler,[†] Holger Arthaber,[‡] Robert Winkler,[¶] Robert G. West,[†] Ioan Ignat,[†] Harald Plank,[¶,§,‖] and Silvan Schmid[*,†]

†*Institute of Sensor and Actuator Systems, TU Wien, Gusshausstrasse 27-29, 1040 Vienna, Austria.*

‡*Institute of Electrodynamics, Microwave and Circuit Engineering, TU Wien, Gusshausstrasse 25, 1040 Vienna, Austria.*

¶*Christian Doppler Laboratory for Direct-Write Fabrication of 3D Nanoprobes (DEFINE), Institute of Electron Microscopy and Nanoanalysis, Graz University of Technology, Steyrergasse 17, 8010 Graz, Austria.*

§*Institute of Electron Microscopy and Nanoanalysis, Graz University of Technology, Steyrergasse 17, 8010 Graz, Austria.*

‖*Graz Centre for Electron Microscopy, Steyrergasse 17, 8010 Graz, Austria.*

E-mail: silvan.schmid@tuwien.ac.at

Supporting Data

S1: Theory of the optical detection method

We used the optical detection scheme presented by Molina et al.[1] to detect the motion of the wide pillar and mapped the amplitude of the optical signal as function of the laser position for two frequencies. The results are presented in the main text. In contrast to Molina et al.,[1] the diameter of our pillar is wider than the spot size of the gaussian beam. For this reason, we can not directly compare our measured amplitude maps with the results of Molina et al.[1] However, Molina et al.[1] gave a theoretical model for the generation of the optical signal, which we used for comparison. The result of the applied model can be seen in Supplementary Fig. 1a-c. Supplementary Fig. 1a shows the total power of the reflected light when the pillar is not actuated. For this measurement, we used an APD410A/M DC avalanche photodiode from THORLABS. Having in mind that the pillar has a diameter of 2.2 µm, it can be seen

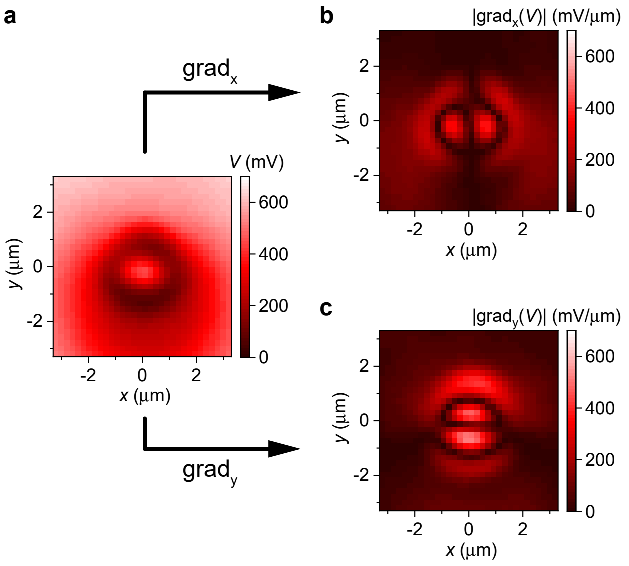

Supplementary Figure 1: Optical detection model. (a) Total power of the reflected light, given by the output voltage V of the photodiode used for detection, as a function of the laser position. The pillar is located around the center of the map and is not actuated. (b), (c) Gradients of the reflected power along the x- and y-axis, which correspond to the vibration axes of the pillar.

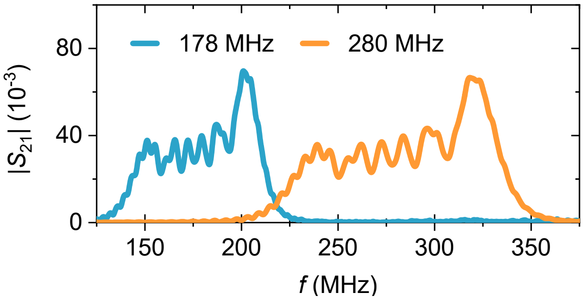

Supplementary Figure 2: Frequency response of two devices with interdigital transducers (IDTs) of different central frequency. The two IDTs of a device face each other and were designed to be equivalent to the orthogonally arranged IDTs discussed in the main text, taking into account the anisotropy of the substrate.

that the pillar mainly scatters the incident light at its edge. Based on the model of Molina et al., the maps of the optical amplitude signal are given by the gradient of the total reflected power along the vibration axis of the pillar. The gradients of the reflected power are given in Supplementary Fig. 1b,c and agree well with the measured amplitude maps shown in the main text. Hence, the optical signal seems to be mainly generated as predicted by the model of Molina et al.[1]

S2: Transmission scattering parameter of the IDTs

In the main text, we present an equivalent circuit model for the SAW transduction scheme. The model treats each of the IDTs as a single two port network, which is described by scattering parameters. To determine the transmission scattering parameter of the IDTs in the equivalent circuit model, we measured two IDTs facing each other. The IDTs were placed along the crystallographic X-axis of the lithium niobate ($LiNbO_3$) substrate and were designed to be equivalent to the orthogonally arranged IDTs used for the pillar measurements discussed in the main text, taking into account the anisotropy of the substrate.

The transmission scattering parameter $|S_{21}|$ of two devices with facing IDTs are shown in Supplementary Fig. 2 as a function of frequency: one device with a central frequency of 178 MHz and another with a central frequency of 280 MHz, like the devices discussed in the

main text. Since we measured the transmission scattering parameter of the overall device, the transmission scattering parameter of the IDTs S_{IDT} is related to S_{21} by

$$S^2_{\mathrm{IDT}}(f) = S_{21}(f)\ . \tag{1}$$

Methods

S3: Device fabrication

Fig. 1b in the main text shows a SEM image of one of the devices used in this study. The substrate is black $LiNbO_3$ with a 128° Y-cut orientation and a thickness of $(350 \pm 20)\,\mu m$. On top of the substrate, we fabricated interdigital transducers (IDTs) and a pillar resonator. The IDTs were structured by standard UV lithography and deposited by thermal evaporation of Ti(5 nm)/Al(150 nm)/Au(5 nm). The shape of the electrodes of the IDTs are designed to match the wave surface of the SAWs emitted by the pillar.[2–4] We calculated the wave surface based on the results of Kovacs et al.,[5] which we also used to determine the chirped distances between the electrodes of the IDTs.[6] The IDTs are placed in such a way that the symmetry axis of an IDT is either parallel or perpendicular to the crystallographic X-axis of the $LiNbO_3$ substrate, which have different phase velocities for SAWs. As a result, the two IDTs of a device differ in length, as shown in Supplementary Table 1. Due to the anisotropy of the $LiNbO_3$ substrate, the electromechanical coupling coefficient between the IDTs and the SAWs is a function of the SAW propagation direction. The coupling coefficient is more or less constant in the range of $\pm 17.5\,\mathrm{deg}$ around the symmetry axes of the IDTs.[7] For this reason, each of the two IDTs covers an angle of 35 deg. However, a wider angle might result in a stronger signal of S_{21}. The distance between the IDTs and the pillar is around 25 wavelengths at the IDTs' central frequency. We chose this distance as a compromise between total size of the device, signal strength and crosstalk between the IDTs.

The pillar resonators are fabricated by Focused Electron Induced Deposition (FEBID)

Supplementary Table 1: Length of the IDTs. The length of an IDT l_{IDT} with central frequency f_{c} is measured along its symmetry axis, which is either parallel ($||$) or perpendicular ($\perp$) to the crystallographic X-axis of the lithium niobate substrate. In addition, the phase velocity of SAWs along the discussed axes are given.

direction	c_{SAW} (m/s)	f_{c} (MHz)	l_{IDT} (µm)
$||$ X	3978	280	691
$\perp$ X	3673	280	638
$||$ X	3978	178	1094
$\perp$ X	3673	178	1010

using a Quanta 3D FEG dual beam microscope from Thermo Fisher Scientific equipped with a standard gas injection system. The FEBID technique is based on gaseous precursor molecules which are locally dissociated on the substrate surface by a focused electron beam. We used $\mathrm{MeCpPt^{IV}Me_3}$ (CAS: 94442-22-5) as precursor molecule, which we heated to 45° for at least 30 min before deposition. The pillars were deposited at a primary beam energy of 5 keV and a beam current of 92 pA by a multi-pass, edge-rounding correcting writing pattern towards the gas flux for optimized pillar shape.[8]

S4: SAW transduction

We used a N5247A PNA-X network analyzer from Keysight for the SAW transduction measurements, which were on wafer calibrated. We applied an input power of −5 dBm and set an IF bandwidth of 10 kHz. The output signal generated by the scattered SAW was superimposed by electrical crosstalk between the two IDTs. The SAW signal travels with around 4000 m/s and is much slower than the electrical crosstalk signal.[5] This allowed us to perform a time gating to remove the electrical crosstalk from the output signal.

S5: Optical detection

We used a UHF lock-in amplifier from Zurich Instruments to conduct the optical detection measurements. We applied a voltage of 750 mV to the emitter IDT to drive the pillar

resonator by SAWs. The optical setup for detection of the pillar's motion is shown in Fig. 2a in the main text. The laser beam was emitted by a TopMode diode laser from Toptica Photonics at a wavelength of 633 nm, incident on the sample with a radiant flux of 76 µW. We focused the laser beam by an objective (x50) with a long working distance on the surface of the substrate resulting in a spot size of the laser beam of around 1.3 µm. The light reflected from the sample was detected by an APD210 avalanche photodidode from MenloSystems with an AC coupled output, which was connected to the input of the lock-in amplifier.

S6: FEM simulations

We performed the FEM simulations in COMSOL Multiphysics (Version 5.5) as described by Kähler et al.[9] except for the meshing of the inner part of the substrate. We applied a maximum mesh element size of an eight of the SAW's wavelength for the whole inner substrate. The reason for this is the smaller size of our geometry in comparison to the pillar pair simulated by Kähler et al.[9]

Supplementary References

(1) Molina, J.; Ramos, D.; Gil-Santos, E.; Escobar, J. E.; Ruz, J. J.; Tamayo, J.; San Paulo, á.; Calleja, M. Optical Transduction for Vertical Nanowire Resonators. *Nano Letters* **2020**, *20*, 2359–2369.

(2) Laude, V.; Gérard, D.; Khelfaoui, N.; Jerez-Hanckes, C. F.; Benchabane, S.; Khelif, A. Subwavelength focusing of surface acoustic waves generated by an annular interdigital transducer. *Applied Physics Letters* **2008**, *92*, 1–3.

(3) Benchabane, S.; Salut, R.; Gaiffe, O.; Soumann, V.; Addouche, M.; Laude, V.; Khelif, A.

Surface-Wave Coupling to Single Phononic Subwavelength Resonators. *Physical Review Applied* **2017**, *8*, 1–7.

(4) O'Rorke, R.; Winkler, A.; Collins, D.; Ai, Y. Slowness curve surface acoustic wave transducers for optimized acoustic streaming. *RSC Advances* **2020**, *10*, 11582–11589.

(5) Kovacs, G.; Anhorn, M.; Engan, H.; Visintini, G.; Ruppel, C. Improved material constants for LiNbO3 and LiTaO3. *IEEE Symposium on Ultrasonics* **1990**, 435–438.

(6) Morgan, D. *Surface Acoustic Wave Filters*, 2nd ed.; Elsevier Ltd., 2007.

(7) Zhang, N.; Mei, J.; Gopesh, T.; Friend, J. Optimized, Omnidirectional Surface Acoustic Wave Source: 152° Y-Rotated Cut of Lithium Niobate for Acoustofluidics. *IEEE Transactions on Ultrasonics, Ferroelectrics, and Frequency Control* **2020**, *67*, 2176–2186.

(8) Winkler, R.; Szkudlarek, A.; Fowlkes, J. D.; Rack, P. D.; Utke, I.; Plank, H. Toward ultraflat surface morphologies during focused electron beam induced nanosynthesis: Disruption origins and compensation. *ACS Applied Materials and Interfaces* **2015**, *7*, 3289–3297.

(9) Kähler, H.; Platz, D.; Schmid, S. Surface acoustic wave coupling between micromechanical resonators. *Communications Physics* **2022**, *5*, 1–8.
